# Recurrent Ipsilateral Facial Nerve Paralysis With Numbness in a 12-Year-Old Girl: A Case Report and Review of Pediatric Recurrence

**DOI:** 10.7759/cureus.105967

**Published:** 2026-03-27

**Authors:** Wyatt C Mayer, Louise N Mendoza, Mario Loomis

**Affiliations:** 1 Department of Clinical Anatomy, Sam Houston State University College of Osteopathic Medicine, Conroe, USA

**Keywords:** bell's palsy, case report, facial numbness, otolaryngology, pediatrics, recurrent facial palsy

## Abstract

Bell's palsy, an idiopathic facial nerve paralysis (FNP), causes one-sided facial muscle weakness. Although this is a common clinical diagnosis in the pediatric population, further workup is warranted if FNP is accompanied by atypical symptoms or recurrence as this could be indicative of an alternative diagnosis. We report a 12-year-old female patient who experienced two right-sided episodes of lower motor neuron FNP occurring 10 months apart with atypical sensory nerve involvement. Both episodes resolved with oral corticosteroid therapy, and the patient did not follow up with pediatric neurology for further workup. This case is notable for its recurrent ipsilateral presentation in a child with otherwise normal systemic workup, contributing to the limited literature on pediatric recurrence patterns. It underscores the diagnostic challenge of distinguishing idiopathic from secondary causes, reinforces the role of corticosteroids in management, and highlights the importance of clinical follow-up despite spontaneous recovery.

## Introduction

Bell's palsy, the idiopathic form of facial nerve paralysis (FNP), is characterized by unilateral weakness of facial muscles innervated by cranial nerve VII [1-3]. Physical signs include loss of mobility of the ipsilateral eyebrow, unilateral incomplete lid closure, drooping of the corner of the mouth, and dry eye [1-3]. Understanding the anatomy of the facial nerve is important to appreciate the different presentations of FNP. The facial nerve travels from the internal acoustic meatus into the facial canal of the temporal bone, where mild inflammation can cause dysfunction and Bell's palsy. It then exits the geniculate ganglion and middle ear, where infections can affect adjacent branches prior to its exit from the skull through the stylomastoid foramen. Insults after this point cause pure motor defects [1,3]. Impaired taste and periauricular pain can be present if the facial nerve is affected more proximally [1,3]. Parotid gland swelling can cause branch-specific deficits of the facial nerve. Typical FNP is primarily a clinical diagnosis without need for imaging or lab work [1,3].

The incidence of FNP in the pediatric population varies by age group, with 2.7 cases per 100,000 in children younger than 10 years of age and 10.1 cases per 100,000 in children between 10 and 20 years of age [1]. Prognosis is excellent in pediatric patients with a recovery rate of approximately 97% [1,2]. Some studies report a similarly high recovery rate even without steroid therapy [1,2,4]. Incomplete recovery without steroid use is more common in adult populations, seen in approximately 20% of patients [1,3].

Recurrent FNP is especially uncommon in children [1,2,5]. Some studies report recurrence from as low as 6% to as high as 14% of cases, although most report rates under 10% [1,5]. Although FNP has an excellent prognosis, as high as 50% of cases can have slight asymmetry which can cause significant social distress in children [1,5]. Because of this risk, scheduled follow-up visits with a pediatrician or specialist through the resolution of symptoms are recommended [1,5].

FNP warrants further evaluation for alternative etiologies when the presentation is suggestive of multi-nerve involvement or when recurrence is accompanied by new or progressive symptoms. We present an atypical case of recurrent pediatric FNP and review current diagnostic and management strategies. In the absence of a standardized, evidence-based algorithm, this case underscores critical gaps in clinical guidance and offers practical insights to support a more structured approach to evaluation and management. 

## Case presentation

A 12-year-old Caucasian female patient from Southeast Texas presented to the clinic in September of 2025, with facial swelling on the right side and pain, notably behind the ear and radiating down the jaw. She also now reports some right-sided numbness. Her symptoms began two weeks prior, initially with pain and swelling alone. Facial weakness and asymmetry developed a few days after the onset of facial swelling. She denied any preceding illnesses or facial trauma. She denied any fever, cough, congestion, sore throat, or tragal tenderness.

**Figure 1:**
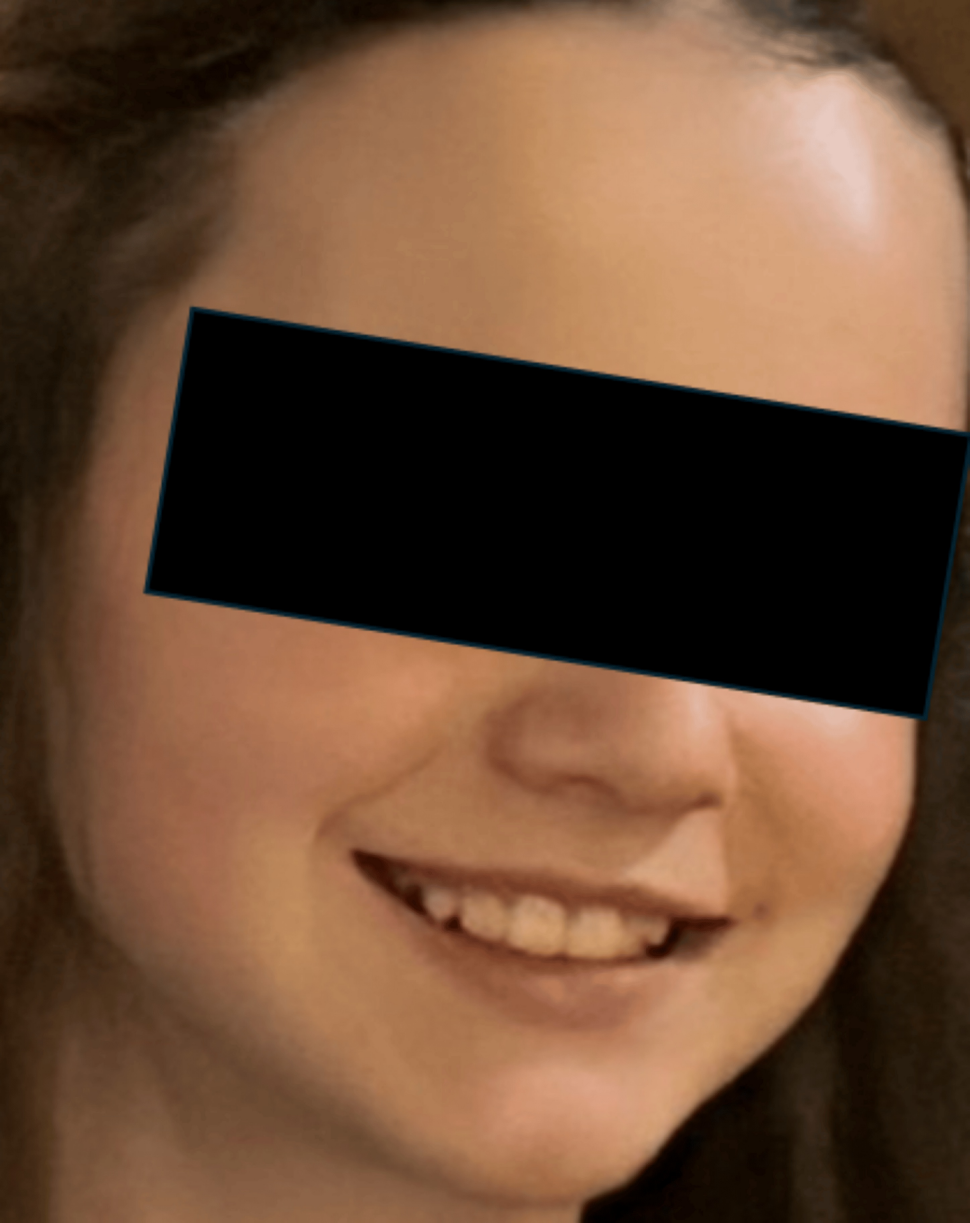
Resolution of symptoms following treatment of the initial episode

The patient was brought in for treatment to her established pediatrician, who she saw for a previous episode in November of 2024 that resolved shortly following oral prednisone therapy (20 mg BID ×3 days followed by 20 mg QD ×3 days). The first incident included not only right-sided facial drooping and asymmetry three days after the onset of right ear pain but also an erythematous tympanum and a lack of sensory loss. Evaluation included a comprehensive metabolic panel and complete blood count; all results were within normal reference ranges. The patient was treated with oral prednisone and deferred follow-up of this complaint due to the resolution of symptoms (Figure 1).

This patient has moderate obesity but was otherwise healthy with no known chronic diseases or infections and takes no chronic medications. She was a student in the sixth grade; her symptoms did not negatively impact her performance in school. There were no sick contacts at school or home, and she denied any recent travel.

**Figure 2:**
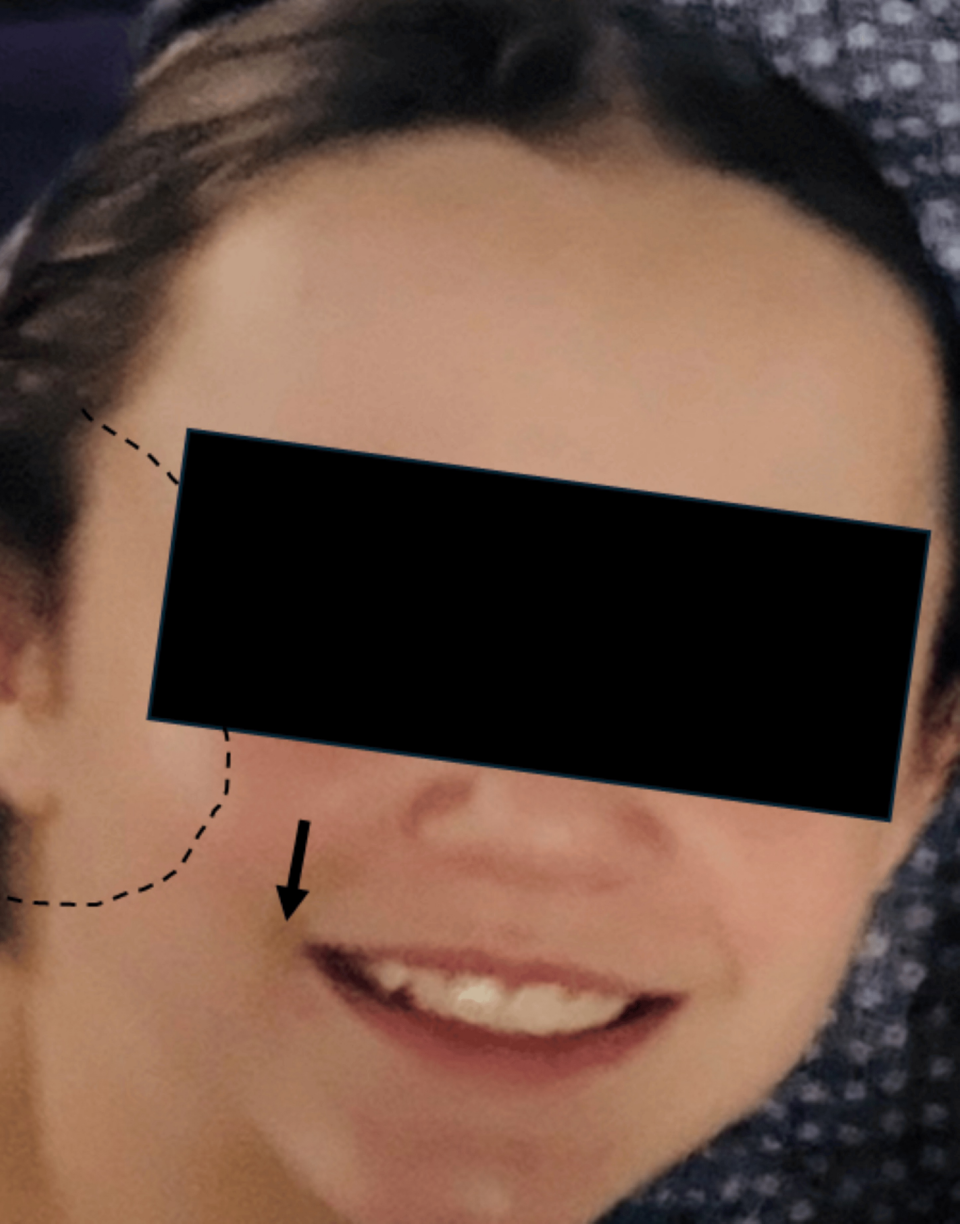
Appearance before the second clinic visit showing mild depression of the right oral commissure (arrow) compared with Figure 1 and the approximate region of facial hypoesthesia (dotted line)

**Figure 3:**
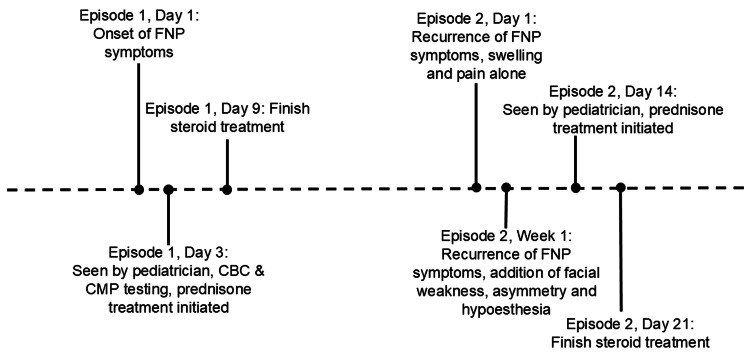
Chronological overview of symptom development, diagnostic evaluation, and treatment FNP: facial nerve paralysis; CBC: complete blood count; CMP: comprehensive metabolic panel

Her review of systems was unremarkable. Vitals were within normal limits aside from tachycardia (heart rate (HR): 120 bpm). During examination, she had facial asymmetry and weakness on the right side, as well as clear right eye discharge. The patient had decreased sensation to light touch and pinprick over the maxillary and mandibular distribution of the trigeminal nerve (Figure 2). The inability to fully raise the eyebrow was present unilaterally. There were no hearing changes, rashes, or abnormal findings on otoscopic examination. The patient was prescribed oral steroids again (prednisone 20 mg BID ×3 days followed by 20 mg QD ×3 days); lab work was not repeated (Figure 3). A referral to a pediatric neurologist was made; however, the patient was lost to follow-up. 

Follow-up and outcomes

The patient did not return for scheduled follow-up appointments, and further diagnostic evaluation could not be completed.

## Discussion

While recurrent idiopathic facial palsy has been described in adults, pediatric recurrence, particularly on the same side, remains uncommon and poorly characterized in the literature [1,5]. Therefore, recurrent FNP in the pediatric population should warrant further investigation into etiologies beyond idiopathic Bell's palsy. Isolated cases in the pediatric population are usually well tolerated. Recurrence of symptoms, however, can lead to long-term cosmetic and quality of life issues and can be the sign of an underlying pathological sequelae [5].

Several conditions have been linked to FNP in pediatrics. These conditions can be categorized into central and peripheral sources (Table 1). Bell's palsy is a peripheral source and the most common overall pathology [1,2,4]. Non-idiopathic causes of peripheral and central FNP include infectious, congenital, traumatic, or malignant sources. Bilateral FNP may be caused by disorders such as sarcoidosis, leukemias, malignancies, Lyme disease, or intracranial masses [2,4,6,7]. Recurrent unilateral FNP should prompt investigation for herpes simplex latency, varicella-zoster infection, congenital facial canal narrowing, or intracranial neoplasms [2,4]. The underlying pathology of these conditions involves either a local inflammatory or mass effect on cranial nerve VII. Recurrent laterality, especially with progressive or new symptoms, is a further indication for clinicians to investigate for a non-idiopathic cause [2,4,5]. The development of unilateral sensory loss in this patient's second episode is especially concerning, as it suggests the presence of multiple nerve involvement (cranial nerves V and VII). This case's absence of systemic symptoms supports a localized pathophysiology; however, these additional findings broaden the differential to include processes affecting the cavernous sinus, skull base, or meninges.

**Table 1:** Differential diagnoses of pediatric facial nerve paralysis by central and peripheral etiologies HSV: herpes simplex virus; CNS: central nervous system; CSF: cerebrospinal fluid Table Credit: Wyatt C. Mayer

Etiological category	Central causes	Peripheral causes	Key diagnostic clues
Infectious	Brainstem encephalitis (e.g., HSV, enterovirus), meningitis, neuroborreliosis with CNS involvement, tetanus, cytomegalovirus, poliomyelitis, human immunodeficiency virus	Lyme disease (*Borrelia burgdorferi*), HSV, varicella-zoster virus, Epstein-Barr virus, botulism, acute otitis media, mastoiditis	Fever, headache, rash, tick exposure, otalgia, vesicular lesions, CSF abnormalities
Inflammatory/autoimmune	Neurosarcoidosis, demyelinating disease (e.g., pediatric multiple sclerosis)	Sarcoidosis, Guillain-Barré syndrome, Melkersson-Rosenthal syndrome	Bilateral or recurrent episodes, other cranial neuropathies, facial edema, fissured tongue, systemic findings
Neoplastic	Brainstem glioma, posterior fossa tumors, leukemia (brain/brainstem infiltration)	Facial nerve schwannoma, parotid gland tumors, temporal bone neoplasms, leukemia	Progressive or recurrent symptoms, incomplete recovery, hearing loss, facial twitching, constitutional symptoms, multiple cranial nerves affected
Congenital/structural	Brainstem malformations	Congenital facial canal stenosis, temporal bone anomalies, drug-induced (i.e., maternal thalidomide use)	Early onset, recurrent episodes from childhood, imaging abnormalities
Traumatic/iatrogenic	CNS injury	Temporal bone fracture, surgical or procedural injury, post-traumatic edema, head entrapment	History of head trauma, otologic surgery, or birth-related injury
Vascular	Arteriovenous malformation, intracranial hypertension	Hemangioma involving the facial nerve, embolization	Acute onset with focal neurologic deficits, imaging evidence of vascular lesions
Idiopathic	N/A	Bell's palsy (recurrence uncommon in children)	Acute onset, unilateral weakness, absence of systemic or neurologic red flags
Metabolic	N/A	Diabetes mellitus, acute porphyria	Progressive onset, usually with peripheral symptoms. Other associated metabolic symptoms present

In such cases of recurrent FNP, further evaluation is recommended. This usually includes magnetic resonance imaging of the brain and auditory canals and laboratory testing as indicated by clinical symptoms and local epidemiological factors. Imaging is essential for assessing central nervous system or structural causes [1]. Laboratory testing and serology can help to identify leukemias and autoimmune and infectious etiologies [1]. Involving pediatric specialists such as otolaryngology and neurology can help to improve the comprehensiveness of the evaluation [1,4]. Unfortunately, the patient in this case was lost to follow-up prior to evaluation by a neurologist. The diagnostic findings are therefore limited as is our ability to determine the underlying etiology.

Treatment for the recurrence of FNP has not been thoroughly investigated; therefore, pediatric patients typically receive the same course of corticosteroids after the primary episode [1]. The efficacy of this treatment is questionable in pediatrics, with many patients recovering from isolated FNP with placebo therapy alone [1]. Antiviral therapy is not as common in pediatric FNP as it is in the adult population; however, children show improved rates of resolution on antiviral therapy if a viral etiology is suspected [1]. In this case, the patient had self-reported resolution of symptoms after oral prednisone therapy, supporting an inflammatory process.

Loss to follow-up represents a significant challenge in pediatrics as it may result in delayed or even missed diagnoses. The absence of continued clinical observation of these cases can interfere with progressive or treatable conditions. This is especially concerning for conditions with intermittent or self-resolving symptoms, as is the scenario for the patient in our case. Even if recurrent FNP is due to Bell's palsy, long-term full recovery is limited to 68-78% of patients with some patients being unaware of their long-term symptoms (i.e., slight facial asymmetry) [5]. This case highlights the importance of counseling patients and their caregivers about the need for follow-up evaluation. They should be aware of the potential causes of recurrent FNP and the importance of following up, even in cases where initial symptoms resolve [3,8]. The focus on patient education can help avoid long-term complications and the possibility of overlooking an early sign of an underlying pathology [3,9]. This emphasis should be especially stressed in cases where the subtle progression of symptoms points towards a non-idiopathic origin.

The limitations of this case include the absence of longitudinal outcome data and an incomplete diagnostic investigation. Despite this, this report reinforces the value of conservative management and careful follow-up while underscoring the need for standardized diagnostic pathways to differentiate idiopathic recurrence from secondary causes of FNP. This report also emphasizes the importance of patient education once recurrence occurs. Without adequate education, patients are easily lost to follow-up once any noticeable symptoms resolve as is the case with the patient from this report. Further reports and long-term follow-up studies would aid in the clarification of the mechanism of recurrence. This would improve care for this rare pediatric presentation.

## Conclusions

This case underscores that recurrent idiopathic facial nerve palsy, while rare in pediatrics, can present ipsilaterally and respond favorably to corticosteroid therapy. The rarity of ipsilateral recurrence in children provides insight into potential localized pathophysiologic mechanisms and the need for standardized evaluation algorithms in this population. Despite a favorable prognosis, clinicians should maintain vigilance for underlying etiologies and consider imaging if recurrence or atypical symptoms (multiple nerves or bilaterality) are involved. Patient and caregiver education should be emphasized in such cases so as to avoid losing patients to follow-up and potentially missing an underlying diagnosis.
